# Effect of Perineum Block Anesthesia Combined with Unprotected Perineal Delivery on the Perineal Integrity Rate and Maternal-Infant Outcomes in Primiparas Taking Health Products Containing Traditional Chinese Medicine

**DOI:** 10.1155/2021/2102618

**Published:** 2021-07-01

**Authors:** Xu Liu, Jianyun Ge, Jiejie Zhang, Boxiang Du

**Affiliations:** Department of Anesthesiology, The Second Affiliated Hospital of Nantong University (Nantong First People's Hospital), Nantong 226001, Jiangsu Province, China

## Abstract

**Objective:**

The purpose of the study was to investigate the effect of perineum block anaesthesia combined with unprotected perineal delivery on the perineal integrity rate and maternal-infant outcomes in primiparas taking health products containing traditional Chinese medicine (TCM).

**Methods:**

A total of 120 puerperae admitted to our hospital from July 2019 to July 2020 were selected as study subjects and divided into group A (*n* = 60) and group B (*n* = 60), according to the number table method. Both groups took health products containing TCM, and the puerperae in group A received perineum block anaesthesia combined with unprotected perineal delivery, while those in group B were treated with routine delivery combined with routine protected perineal delivery. After that, the effect of different delivery modes on the perineal integrity rate and maternal-infant outcomes in puerperae was analyzed by the comparison of delivery condition, perineal condition, and postpartum quality of life between the two groups.

**Results:**

There were no significant differences in average age and other general data between the two groups (*P* > 0.05); the duration in first, second, and third stages of labor in group A was significantly lower than that in group B (*P* < 0.001); the Apgar score in group A was significantly higher than that in group B (*P* < 0.001); the number of puerperae with integrated perineum in group A was significantly higher than that in group B (*P* < 0.05), while the number of puerperae receiving episiotomy in group A was significantly lower than that in group B (*P* < 0.05); the quality of life score in group A was significantly higher than that in group B (*P* < 0.001); the incidence of maternal postpartum complications in group A was significantly lower than that in group B (*P* < 0.05).

**Conclusion:**

Perineum block anaesthesia combined with unprotected perineal delivery can effectively shorten maternal labor duration, improve perineal integrity rate, and reduce laceration of perineum, with a significant therapeutic effect, which is worthy of application and promotion.

## 1. Introduction

Natural delivery is a physiological process during which both mother and newborn actively participate in and complete the delivery, making it the optimal delivery mode [1], and in the process of natural delivery, maternal regular uterine contraction and birth canal extrusion can promote rhythmic contractions in fetal thoracic cavity, so that the pulmonary function of newborns can be exercised to induce their spontaneous breathing. In addition, fetal algesia, gustation, and sense of touch can be stimulated in the birth canal, thus promoting the development of the brain and vestibular function area and benefiting later movement and the formation of personality; therefore, the natural delivery proves to be the most effective delivery mode to improve birth quality [2, 3]. Health products with traditional Chinese medicine (TCM) ingredients such as Yunkang Granules can effectively adjust the body of women during pregnancy, have a unique effect on the treatment of recurrent abortion, and promote fetal health. Cesarean section can adversely affect maternal recovery and later neonatal development and subsequent pregnancy, eventhough it shows an improvement in maternal delivery safety to some extent. Clinical studies have confirmed that supine delivery affects maternal uterine blood supply and prolongs the duration in the second stage of labor, increasing the risks of episiotomy and dystocia. Episiotomy, a type of midwifery operation for smooth delivery, refers to the procedure of making an oblique incision in maternal perineum, so as to avoid perineal laceration and protect pelvic floor muscles [4–6]. Perineum block anaesthesia, serving the functions of relieving labor pain and reducing the risks of perineal laceration, has been widely used in obstetrics and has brought a boon to the majority of puerperae. Generally, traditional midwifery is performed by implementing artificial perineal dissection for puerperae, which can avoid perineal laceration occurring in puerperae during delivery but leaving pathological incisions affecting postpartum recovery; however, unprotected perineal delivery is aimed at reducing the rate of cesarean section, relieving maternal labor distress, and returning delivery to nature [6, 7]. Based on this, this study aimed to investigate the effect of perineum block anaesthesia combined with unprotected perineal delivery on the perineal integrity rate and maternal-infant outcomes in primiparas taking health products containing TCM, reported as follows.

## 2. Materials and Methods

### 2.1. General Information

A total of 120 puerperae admitted to our hospital from July 2019 to July 2020 were selected as study subjects and divided into group A (*n* = 60) and group B (*n* = 60), according to the number table method. This study was approved by the Hospital Ethics Committee, and the puerperae and their family members were informed of the purpose and process of this study and signed the informed consent.

### 2.2. Inclusion Criteria

Puerperae were all primiparas with singleton pregnancy. Puerperae had delivery certificate. Puerperae had no contraindications to perineum block anaesthesia.

### 2.3. Exclusion Criteria

Puerperae had abnormal fetal position, macrosomia, or placental abruption. Puerperae had organic lesions in the brain, heart, and liver. Puerperae had perineal inflammation, scars, and others seriously affecting perineal delivery. Puerperae had the history of pelvic surgery.

### 2.4. Methods

The puerperae in both groups before delivery orally took Yunkang Granules (NMPA approval no: Z19991100; manufacturer: Jilin Aodong Pharmaceutical Group Co., Ltd., Dalian Branch; specification: 8 g∗9 bags) on an empty stomach in the morning, at noon, and in the evening, 1 bag/time, 3 times/day, for 15 days continuously.

The puerperae in group B were treated with routine delivery combined with routine protected perineal delivery. During surgery, after maternal uterine orifice was fully opened, the puerperae with supine positions on an obstetric table were instructed to rely on the rhythmic contractions of uterine to push the fetus out of the body, and meanwhile, 4 conventional surgical cotton pads were used to protect the maternal perineum [8, 9].

The puerperae in group A received perineum block anaesthesia combined with unprotected perineal delivery, and specific steps were as follows. After maternal uterine orifice was fully opened, the perineum block anaesthesia was performed in the way that 10 ml of 2% lidocaine hydrochloride injection (State Food and Drug Administration approval number: H61023138; manufacturer: Shaanxi Jianmin Pharmaceutical Co., Ltd.; specification: 5 ml: 50 mg) was taken by a syringe and then diluted with 10 ml of normal saline solution to 20 ml. After that, doctor's middle finger and forefinger entered into maternal vagina and could not stop until touching ischial spine, and then, puncture was conducted into the site about 0.5 cm from the inner side of the ischial spine with number 9 puncture needle along the surgical direction. After confirming that there was no blood when the needle was drawn back, the puerperae were injected with 5 ml of lidocaine, and then, the needle was withdrawn as the injection was slowly carried out. The remaining 5 ml of lidocaine was used for perineal local infiltration anaesthesia. During the process of unprotected perineal delivery, puerperae first took nonsupine positions and then took semireclining positions after the total opening of uterine orifice. When 2-3 cm of the fetal head was visible on vulval gapping, delivery was ready, and at the same time, with paying much attention to slowing the rate of fetal head delivered, medical staff should instruct the puerperae to practise abdominal pressure correctly and guide them to cooperate with breathing, so as to ensure smooth delivery.

### 2.5. Observation Indexes

The labor duration and maternal postpartum complications were recorded and compared between the two groups.

The neonatal conditions in both groups were evaluated by the Apgar [10] scale, with the total score of 10 points. The score of 8–10 points represented normal condition, the score of 4–7 points represented mild asphyxia, and the score of 0–3 points represented severe asphyxia.

#### 2.5.1. Evaluation Criteria

First-degree laceration of perineum referred to that there was slight laceration occurring between perineal epidermis and vaginal mucosa, and there was no injury in the muscle layer. Second-degree laceration of perineum laceration referred to that laceration occurred in the perineal skin, mucosa of posterior vaginal wall, and muscle layer of vaginal mucosa. Third-degree laceration of perineum laceration referred to that there was severe laceration, involving the rupture of sphincter ani externa muscle, vaginal, anal, and rectal penetration, and rectal exposure.

The postpartum quality of life in both groups was evaluated by the Maternal Postpartum Quality of Life Rating Scale made by our department, which included the items of mental state, sleep quality, appetite, and daily activity, with each item scoring 5 points, and higher scores indicated better postpartum quality of life.

### 2.6. Statistical Treatment

All the study data were processed for statistical analysis by SPSS21.0 software, and GraphPad Prism 7 (GraphPad Software, San Diego, USA) was used to draw the pictures of the data. Measurement data were expressed by ($\bar{x}$ ± *s*) and tested by the *t*-test. Enumeration data were expressed as (*n* (%)) and tested by the *X*^2^ test. The differences had statistical significance when *P* < 0.05.

## 3. Results

### 3.1. Comparison of Clinical Information between the Two Groups

There were no significant differences in average age, mean premature rupture of membranes time, mean gestational week, mean height, education level, and place of residence between the two groups (*P* > 0.05), as given in Table 1.

### 3.2. Comparison of Maternal Delivery between the Two Groups

The duration in first, second, and third stages of labor in group A was significantly lower than that in group B (*P* < 0.001), as given in Table 2.

### 3.3. Comparison of Apgar Score between the Two Groups

**Figure 1:**
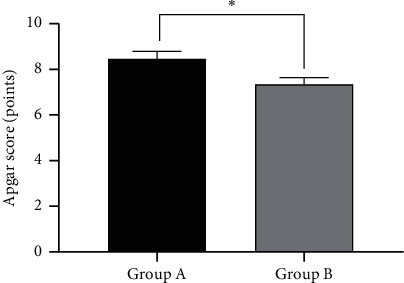
Comparison of Apgar score between the two groups ($\bar{x}$ ± *s*).

The Apgar score in group A was significantly higher A than that in group B (*P* < 0.05), as shown in Figure 1.

The abscissa represented group A and B, while the ordinate represented Apgar score points. The Apgar scores were (8.23 ± 0.46) points in group A and (7.13 ± 0.43) points in group B. *∗* indicated that there were significant differences in Apgar scores between the two groups (*t* = 13.532, *P* < 0.001).

### 3.4. Comparison of Maternal Perineum Condition between the Two Groups

The number of puerperae with integrated perineum in group A was significantly higher than that in group B (*P* < 0.05), while the number of puerperae receiving episiotomy in group A was significantly lower than that in group B (*P* < 0.05); there were no significant differences in the number of puerperae with first-, second-, and third-degree laceration of perineum between the two groups (*P* > 0.05), as given in Table 3.

### 3.5. Comparison of Postpartum Quality of Life Score between the Two Groups

The scores of postpartum mental state, sleep quality, appetite, and daily activity in group A were significantly higher than those in group B (*P* < 0.05), as given in Table 4.

### 3.6. Comparison of Maternal Postpartum Complications between the Two Groups

The incidence of maternal postpartum complications in group A was significantly lower than that in group B (*P* < 0.05), as given in Table 5.

## 4. Discussion

Studies have found that natural delivery can generate a stress response, which is mainly stimulated by fetal birth canal pressure, hence leading to a series of endocrine alterations and promoting the production of neonatal immune factors to enhance immune function, providing more resistance for neonates than cesarean section. In addition, natural delivery can also promote postpartum milk secretion and improve the relationship between puerperae and fetuses [11, 12]. The study found that taking health products containing TCM before delivery could invigorate the spleen and kidney, nourish blood, and prevent abortion, which had a significant effect on pregnant women with habitual abortion. Cesarean section is suitable for puerperae with difficulties in delivery; although it can shorten labor duration, it brings more trauma to the maternal tissues, resulting in slow postpartum recovery. With continuous popularization of health knowledge, there have been more and more puerperae who prefer natural delivery; however, the own limitations of this delivery mode increase the difficulties in the obstetric work, and thus, how to improve the natural delivery rate and shorten the postpartum recovery duration has become the research focus in the medical community [13–15]. Unprotected perineal delivery, belonging to natural delivery, is a newly emergent delivery mode in recent years, which has broken the limitations of cesarean section. Additionally, this delivery mode can minimize maternal labor pain, reduce the risks of intraoperative bleeding and infection, promote rapid recovery of pelvic floor function, reduce sequelae caused by pelvic floor dysfunction, minimize perineal laceration, and improve perineal integrity rate, effectively facilitating delivery [14, 16, 17].

Pain in the second stage of labor is mainly caused by uterine contractions and forced distension of the pelvic floor during natural delivery, whereas perineum block anaesthesia can greatly reduce the pain caused by distension of the birth canal and pelvic floor during labor, so as to relax the perineum and vagina and effectively shorten the labor duration in the second stage [18]. In this study, after the primiparas with natural childbirth were treated with perineum block anaesthesia combined with unprotected perineal delivery, their perineal integrity rate was significantly higher than that of the puerperae undergoing the routine delivery and routine protected perineal delivery (*P* < 0.05). The duration in first, second, and third stages of labor in group A was significantly lower than that in group B (*P* < 0.001). Laudi and Peeples [19] have stated in their studies that after the primiparas with natural childbirth are treated with perineum block anaesthesia combined with unprotected perineal delivery, their perineal integrity rate is 23.45%, which is significantly higher than that of 6.12% in the routine group, indicating that perineum block anaesthesia combined with unprotected perineal delivery can improve maternal perineal integrity rate because unprotected perineal delivery can reduce the compression of maternal perineum and relax perineal musculature under the guidance of medical staff, so as to relieve delivery injuries. Clinical practice has confirmed [20] that maternal positions during delivery may affect fetal arterial blood pressure and neonatal growth as well as development to some extent. In the routine delivery mode, a supine position is often adopted, which can increase maternal lumbar curvature and make uterus compress iliac arteries and inferior vena cava, thus affecting the abdominal aorta, leading to decreased uterine blood flow and increasing the risks of fetal distress. However, in unprotected perineal delivery, puerperae usually take semireclining positions, which can beneficially promote engagement of fetal head, decrease the resistance of pelvic floor soft tissues to descending fetal head, and increase the compliance of the fetus in the birth canal [21]. This study revealed that the Apgar score in group A was significantly higher than that in group B (*P* < 0.001), demonstrating that perineum block anaesthesia combined with unprotected perineal delivery can reduce the incidence of neonatal asphyxia and improve delivery safety.

In conclusion, perineum block anaesthesia combined with unprotected perineal delivery can effectively shorten labor duration, improve perineal integrity rate and maternal quality of life in the postpartum period, and reduce the risks of delivery, which is worthy of application and promotion.

## Figures and Tables

**Table 1:** Comparison of clinical information between the two groups.

Types	Group A (*n* = 60)	Group B (*n* = 60)	*χ* ^2^/*t*	*P*
Average age (years old)	27.64 ± 2.31	27.68 ± 2.29	0.095	0.924
Mean premature rupture of membranes time (*h*)	6.03 ± 0.58	6.05 ± 0.61	0.184	0.854
Mean gestational week (weeks)	39.06 ± 0.53	39.08 ± 0.56	0.201	0.841
Mean height (cm)	163.42 ± 3.42	163.46 ± 3.48	0.064	0.950
Education level				
Undergraduate education	18 (30.00%)	20 (33.33%)	0.154	0.695
Secondary education	37 (61.67%)	36 (60.00%)	0.035	0.852
Primary education	5 (8.33%)	4 (6.67%)	0.120	0.729
Place of residence			0.033	0.855
Urban area	29 (48.33%)	30 (50.00%)		
Rural area	31 (51.67%)	30 (50.00%)		

**Table 2:** Comparison of maternal delivery between the two groups ($\bar{x}$ ± *s*, min).

Group	*n*	First stage of labor	Second stage of labor	Third stage of labor
Group A	60	443.63 ± 24.52	54.79 ± 9.83	7.23 ± 1.06
Group B	60	475.36 ± 25.67	67.35 ± 8.76	8.97 ± 1.08
*t*		6.924	7.389	8.907
*P*		<0.001	<0.001	<0.001

**Table 3:** Comparison of maternal perineum condition between the two groups (*n* (%)).

Group	*n*	Integrated perineum	Perineal side cut	First-degree laceration of perineum	Second-degree laceration of perineum	Third-degree laceration of perineum
Group A	60	12 (20.00%)	10 (16.67%)	35 (58.33%)	3 (5.00%)	0 (0.00%)
Group B	60	3 (5.00%)	20 (33.33%)	33 (55.00%)	4 (6.67%)	0 (0.00%)
*X* ^2^		6.171	4.444	0.136	0.152	0.000
*P*		0.013	0.035	0.713	0.697	1.000

**Table 4:** Comparison of postpartum quality of life score between the two groups ($\bar{x}$ ± *s*, points).

Group	*n*	Mental state	Sleep quality	Appetite	Daily activity
Group A	60	3.84 ± 1.05	3.75 ± 0.86	3.64 ± 0.53	3.73 ± 0.63
Group B	60	2.93 ± 0.97	3.21 ± 0.74	2.83 ± 0.49	3.14 ± 0.58
*t*		4.931	3.687	8.692	5.337
*P*		<0.001	<0.001	<0.001	<0.001

**Table 5:** Comparison of maternal postpartum complications between the two groups (*n* (%)).

Group	*n*	Nausea and vomiting	Perineum edema	Puerperal infection	Postpartum hemorrhage	Total incidence
Group A	60	1 (1.67%)	0 (0.00%)	1 (1.67%)	0 (0.00%)	3.33% (2/60)
Group B	60	2 (3.33%)	3 (5.00%)	2 (3.33%)	1 (1.67%)	13.33% (8/60)
*X* ^2^						3.927
*P*						0.048

## Data Availability

The data used to support the findings of this study are available from the corresponding author upon request.
